# High-order epistasis shapes evolutionary trajectories

**DOI:** 10.1371/journal.pcbi.1005541

**Published:** 2017-05-15

**Authors:** Zachary R. Sailer, Michael J. Harms

**Affiliations:** 1 Institute of Molecular Biology, University of Oregon, Eugene, OR, USA; 2 Department of Chemistry and Biochemistry, University of Oregon, Eugene, OR, USA; UNITED STATES

## Abstract

High-order epistasis—where the effect of a mutation is determined by interactions with two or more other mutations—makes small, but detectable, contributions to genotype-fitness maps. While epistasis between pairs of mutations is known to be an important determinant of evolutionary trajectories, the evolutionary consequences of high-order epistasis remain poorly understood. To determine the effect of high-order epistasis on evolutionary trajectories, we computationally removed high-order epistasis from experimental genotype-fitness maps containing all binary combinations of five mutations. We then compared trajectories through maps both with and without high-order epistasis. We found that high-order epistasis strongly shapes the accessibility and probability of evolutionary trajectories. A closer analysis revealed that the magnitude of epistasis, not its order, predicts is effects on evolutionary trajectories. We further find that high-order epistasis makes it impossible to predict evolutionary trajectories from the individual and paired effects of mutations. We therefore conclude that high-order epistasis profoundly shapes evolutionary trajectories through genotype-fitness maps.

## Author summary

A key goal for evolutionary biologists is understanding why one evolutionary trajectory is taken rather than others. This requires understanding how individual mutations, as well as interactions between them, determine the accessibility of evolutionary pathways. We used a robust statistical analysis to reveal interactions between up to five mutations in published datasets, meaning that the effect of a mutation can depend on the presence or absence of four other mutations. Simulations reveal that these interactions strongly shape evolutionary trajectories. These interactions lead to profound unpredictability in evolution, as one cannot use the effect of a mutation in the ancestor to predict its effect later in the trajectory.

## Introduction

Epistasis creates historical contingency, as it means that the effect of a mutation depends on previous substitutions [1–6]. Interactions between pairs of mutations can cause mutations to accumulate in a specific order [1, 4], stochastically open and close pathways [3, 6], and make evolution irreversible [7, 8].

The effects of high-order epistasis—interactions between three or more mutations—on evolution are less well understood than the effects of pairwise epistasis. Statistically-significant high-order epistasis has been observed in multiple genotype-phenotype maps [6, 9–16], even when steps are taken to minimize its contribution to epistasis models [15]. Its magnitude is generally lower than the individual and pairwise epistatic effects of mutations [15]. Several studies have suggested that it can alter evolutionary outcomes [6, 10, 16], but its overall importance for evolution is not well understood. Does high-order epistasis alter evolutionary outcomes? Or are trajectories primarily shaped by the additive and pairwise epistatic effects of mutations?

We set out to assess the effect of high-order epistasis on evolutionary trajectories through experimentally measured genotype-fitness maps. We decomposed these maps into contributions from nonlinear scale, additive effects, and epistasis at different orders ranging from second to fifth. We then calculated “truncated” maps with different orders of epistasis deleted. By comparing the fitness values and probabilities of individual evolutionary trajectories through the truncated maps, we can reveal the extent to which high-order epistasis determines evolutionary outcomes.

## Results

### High-order epistasis is common in all maps

Our first goal was to determine the contributions of each order of epistasis to fitness in six experimentally measured genotype-fitness maps (Table 1). Each map consisted of all possible combinations of 5 mutations (2^5^ = 32 genotypes) in a haploid genome. The mutations in datasets I and IV arose during adaptive, experimental evolution of *E*. *coli*, and occur throughout the genome [19, 28]. The mutations in datasets II and VI each occur in single genes that confer drug resistance in *E*. *coli* and HIV, respectively [1, 29]. The mutations in datasets III and V were introduced randomly into the *A*. *niger* genome [30]. Previous workers characterized components of fitness for each genotype under defined experimental conditions. For two of the datasets (I, and IV), the authors measured relative fitness using competition assays. In two of the datasets (III and V), the authors measured growth rate of each strain. In dataset II, the authors measured minimum inhibitory concentration in the presence of an antibiotic, and from this estimated relative fitness [1]. In dataset VI, the authors measured HIV infectivity in an ex vivo assay, then treated this activity as a proxy for fitness [29]. All genotypes and phenotypes for each dataset are shown in S1 JSON.

**Table 1:** Experimental genotype-phenotype maps used in this study.

ID	genotype	organism	reference
I	scattered genomic mutations	*E. coli*	[19]
II	*β*-lactamase enzyme point mutations	*E. coli*	[1]
III	chromosomes combinations	*A. niger*	[30]
IV	scattered genomic mutations	*E. coli*	[28]
V	chromosomes combinations	*A. niger*	[30]
VI	envelope glycoprotein point mutations	HIV-1	[29]

We previously analyzed four of these datasets, finding small magnitude, but statistically-significant, high-order epistasis in each map [15]. We used this same approach to characterize epistasis in the remaining two maps (S1 Fig, Materials & methods). We sought to account for confounding effects that could lead to spurious epistasis, which would, in turn, lead to spurious effects on evolutionary trajectories. The most important confounding effect is the scale of the map. Models of high-order epistasis sum the effects of mutations and then account for deviation from this expectation by epistasis [12, 17]. But there is no *a priori* reason to assume mutational effects should add: they may multiply or combine on some other nonlinear scale [5, 12, 15, 31]. To account for this, we empirically determined a nonlinear scale for each map using a power-transform, and then used this to linearize each map [15].

We then decomposed the linearized maps into epistatic coefficients using Walsh polynomials [10, 12, 17]. This approach uses the geometric center of the genotype-fitness map as reference state and reveals global correlations in the effects of mutations across the map. Each order of epistasis accounts for variation that is not explained by the sum of all lower-order contributions. For example, third-order coefficients account for any “leftover” variation in the fitness of triple mutants after the first-order (additive) and second-order (pairwise) effects of those mutations are taken into account.

We determined the contribution of each order of epistasis to the total variation in fitness for each dataset by sequentially setting fifth-, fourth-, third-, and second-order epistatic coefficients to zero. We recalculated the fitness of each genotype using each “truncated” model. This is directly analogous to decomposing a sound wave into a sum of frequencies using a Fourier transform [12]. After decomposition, the original sound wave can be approximated by a sum of principal frequencies, followed by a reverse Fourier transform. By selectively including frequencies, one can identify those that contribute most to the final sound wave. Our analysis follows the same logic, approximating fitness (the sound wave) using a collection of epistatic coefficients (sound frequencies).

**Fig 1:**
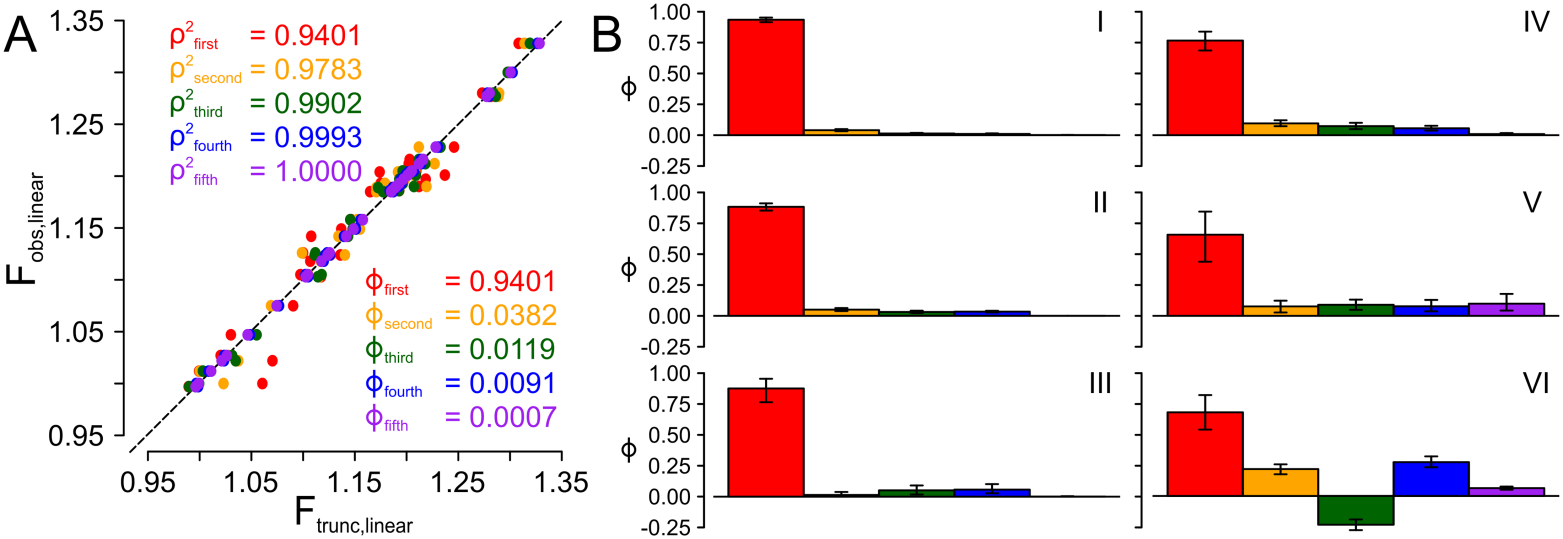
Contributions of epistasis to variation in fitness. Panel A: Correlation between observed (linearized) fitness and fitness calculated for truncated epistasis models for dataset I. Each point on the plot is a single genotype-fitness pair; the dashed line is a 1:1 line. Colors correspond to the truncation order: to first (red), second (orange), third (green), fourth (blue), and fifth (purple). *ρ*^2^ and *ϕ* for each order are shown on the plot, colored by order. Panel B summarizes the contributions of each order of epistasis to the variation in fitness for all six datasets. Dataset is indicated with roman numeral. Colors follow panel A.

We quantified the contribution of each epistatic order by measuring the change in fitness when the *i*^*th*^ order of epistasis was included in the model. As a metric, we used $\phi =\rho_{i}^{2}-\rho_{i-1}^{2}$, where $\rho_{x}^{2}$ is the squared Pearson’s coefficient between the measured fitness of each genotype and its fitness calculated for a model truncated to the *x*^*th*^ order. *ϕ* ranges from -1 to 1. Fig 1A shows this calculation for dataset I. As epistatic orders are added, *ρ*^2^ between the truncated model and measured fitness values improves. This allows determination of *ϕ* for each order: first-order coefficients (additive effects) account for 94.0% of variation in fitness; second-order (pairwise epistasis) for 3.8%; third for 1.2%, fourth for 0.9%, and fifth for 0.1%.

We then applied this analysis to all six datasets. Fig 1B summarizes these results. The total contribution of epistasis to variation in fitness ranged from 6.0% (dataset I) to 32.2% (dataset VI). Other datasets exhibited intermediate levels of epistasis, comparable in magnitude to high-order epistasis observed in similar datasets [10, 15, 16]. In all datasets, the first-order (additive) effects of mutations made the largest contribution to variation in fitness. Outside of this, there was no simple pattern in the relative contributions of the different orders. In dataset I, II and IV, the contribution of epistasis to variation decayed with increasing order. In dataset V, epistasis does not decay. In dataset VI, the addition third-order epistasis (without fourth-order epistasis) actually does a worse job of predicting fitness than second-order alone. The quantitative and qualitative differences in the contribution of epistasis across datasets allow us to study how altering epistasis alters evolutionary trajectories.

### Epistasis alters evolutionary trajectories

Our next question was how each order of epistasis altered evolutionary trajectories. We first back-transformed our truncated, linearized maps onto the original scale. This creates a genotype-fitness map without specific epistatic interactions, but on the original, possibly nonlinear, scale of the map. We calculated the relative probabilities of all *L*! forward trajectories through these maps, starting from the ancestral state and ending at the derived state [1, 19, 30]. Because the maps describe fitnesses of asexual organisms with large population sizes, we modeled trajectories as a series of sequential fixation events captured by a Gillespie model for haploid organisms with large population size (Materials & methods) [1, 18, 19]. In this scheme, the probability of a trajectory is the product of the probabilities of its individual fixation events, normalized across all trajectories.

**Fig 2:**
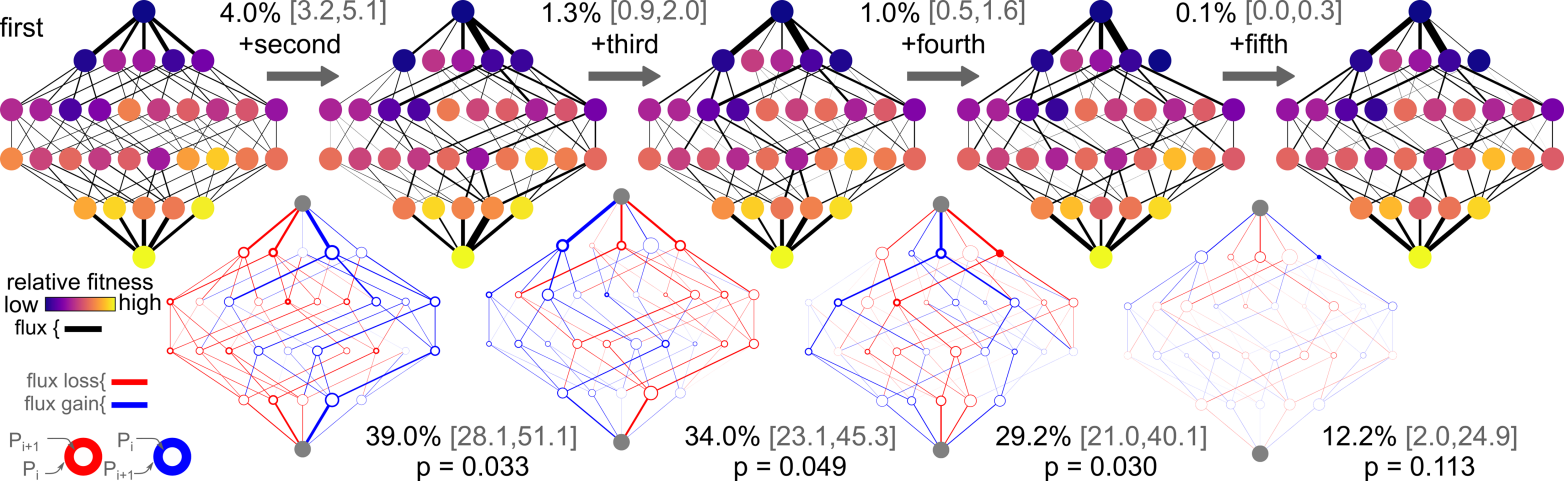
Epistasis alters evolutionary trajectories through genotype-fitness maps. Figure show the effects of increasing orders of epistasis on evolutionary trajectories for dataset I. **Top Row:** Genotype-fitness maps with increasing amounts of epistasis included, increasing from none (far left) to fifth-order (far right). Networks show all 2^5^ genotypes, arranged from ancestral (top) to derived (bottom), colored by relative fitness from low (purple) to high (yellow). Edges show the probability of a given mutation in a given background from low (thin) to high (heavy). Mutations with no probability have no edge. The numbers above the arrows are *ϕ*, with 95% confidence intervals in brackets. **Bottom row:** Change in trajectory probability as each order of epistasis is added. Edges reveal loss of probability (red) or gain of probability (blue). The weight of the edge is directly proportional to the change in probability. Mutations whose probability do not change have no edge. For each node, the thickness of the ring reveals the change in probability that this genotype is visited. For genotypes whose probability goes down, the red area indicates the loss in probability. For genotypes whose probability goes up, the blue area indicates the increase in probability. The numbers below each network are *θ*, with 95% confidence intervals in brackets. The *p*-value measures whether the observed value of *θ* would be expected from fitting experimental noise (Fig 3).

We visualized these trajectories by overlaying them on the genotype-fitness map weighted by their relative probabilities. Higher probability mutations have thicker lines connecting them. Fig 2 shows this analysis for dataset I. We started with a purely additive map (top left). All trajectories are accessible with similar probabilities because, in this map, all mutations are individually favorable. We then added successive orders of epistasis and recalculated trajectories through each new map. The addition of second-order epistasis altered the availabilities of trajectories. The changes are most readily evident in the lower row in Fig 2, which shows the change in the probability of each edge and node in the map. The left side of the map is red (indicating loss of probability), while the right side of the map is blue (indicating gain of probability). Addition of each new order, moving left to right across Fig 2, alters the probability of trajectories through the map.

To quantify differences in the sets of trajectories with increasing epistasis, we calculated the change in the probabilities of all 120 forward trajectories through maps with different amounts of epistasis included (*θ*). A *θ* of 0.0 indicates that the set of trajectories through the spaces are identical, while a *θ* of 1.0 means the sets of trajectories do not overlap at all (Materials & methods). Intermediate values indicate that some fraction of the trajectory probability density is shared between the maps. In dataset I, trajectories through the additive and second-order epistatic maps have *θ* = 0.390. Put another way, the addition of pairwise epistasis to the additive map shifts 39.0% of the trajectory probability density. Addition of each new order of epistasis has a smaller effect on trajectory probability: *θ*_2→3_ = 0.340, *θ*_3→4_ = 0.292, and *θ*_4→5_ = 0.122.

**Fig 3:**
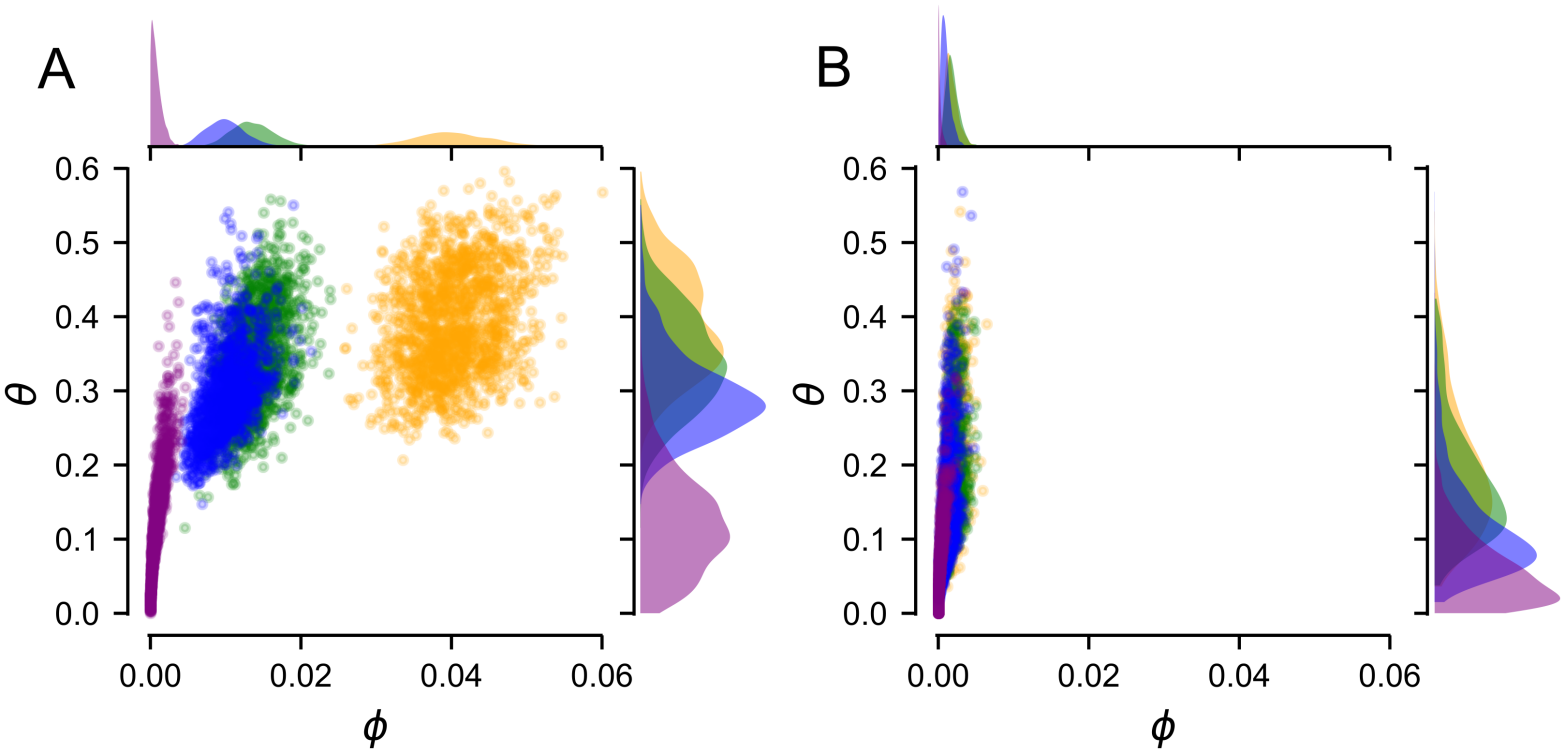
Changes in trajectories are not the result of experimental uncertainty. Data in panel A and B are for dataset I. Panel A shows the distribution of *ϕ* and *θ* for 10,000 pseudoreplicates generated by sampling uncertainty in each the fitness of each genotype (S2A Fig). Colors denote order of epistasis, as in Fig 1. Panel B shows the epistasis extracted from datasets without epistasis, but experimental uncertainty (S2B Fig).

To determine confidence intervals on our estimates of *ϕ* and *θ*, we sampled from the fitness measurement uncertainty for each genotype, generating a collection of pseudoreplicate genotype-fitness maps (S2A Fig). We then decomposed each pseudoreplicate map into epistatic coefficients (including refitting the scale) and remeasured *ϕ* and *θ* for each epistatic order. Fig 3A shows this calculation for dataset I. From these distributions, we can determine 95% confidence intervals for *ϕ* and *θ* (shown as gray, bracketed values in Fig 2).

We next asked whether the observed epistasis and its effect on trajectories could be the result of uncertainty in the fitness values. An epistasis model accounts for random noise as leftover variation, and thus as apparent epistasis [15]. We posed the following question: if the epistasis at a given order resulted only from noise, what effect would it have on *ϕ* and *θ*? To ask this question, we constructed “null” maps with truncated epistasis, but noisy fitness values (S2B Fig). We took our truncated maps at each order and then assigned each fitness the same variance that was measured for the original, un-truncated fitness values. We sampled from this uncertainty to generate pseudoreplicates, extracted apparent epistasis—in this case, arising from noise—and then calculated *ϕ* and *θ* for the pseudoreplicate. This allows us to construct distributions of *ϕ* and *θ* for epistasis arising purely from experimental noise.

We show this calculation for dataset I in Fig 3B. Unlike the experimental distributions, which spread out in *ϕ*, the distributions arising from random noise cluster at low values of *ϕ*. The *ϕ*/*θ* distributions of second-, third-, and fourth-order epistasis minimally overlap in Fig 3A versus 3B. This indicates that the signal for epistasis in the datasets is greater than expected from noise in the measured fitness values. In contrast, the *ϕ*/*θ* distribution for fifth-order epistasis overlaps between Fig 3A and 3B: the effect of fifth-order epistasis cannot be distinguished from noise. Because we are interested in the effect of epistasis on trajectories (*θ*), we determined a *p*-value for each *θ*. We took the mode of *θ* at each order from Fig 3A, and determined its percentile on the corresponding null distribution in Fig 3B. For second-, third-, and fourth-order epistasis, this yields a *p*-value < 0.05. In contrast, the *p*-value for fifth order was 0.12.

With these quantification tools in hand, we next studied the relationship between epistasis and evolutionary trajectories for the increasing levels of epistasis exhibited by the remaining five datasets. S3–S8 Figs summarize our analyses for all six datasets.

**Fig 4:**
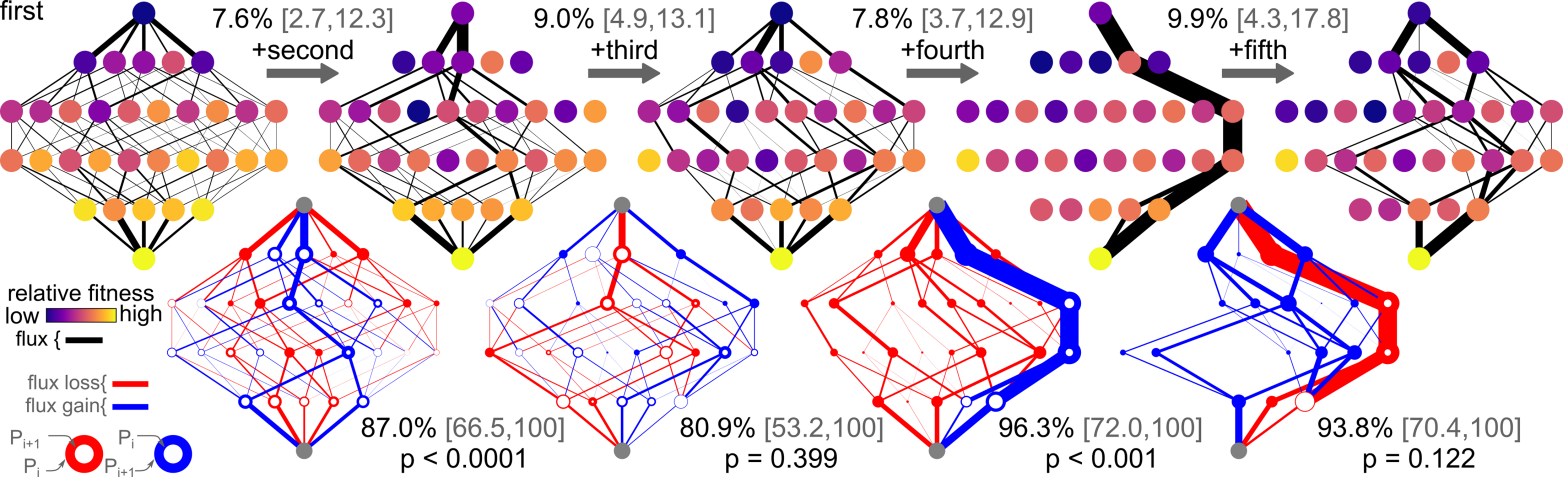
Epistasis alters trajectories in dataset V. Altered trajectories in dataset V with increasing epistasis. Colors, panel layouts, and statistics are as in Fig 2.

It is helpful to compare dataset I (Fig 2) and dataset V (Fig 4). While epistasis accounts for 6.0% of variation in fitness for dataset I, it accounts for 32% of the variation in fitness for dataset V. The large amount of epistasis in dataset V means that epistasis at all orders has a massive effect on evolutionary trajectories through this space. The addition of fourth-order epistasis is particularly striking. With only third-order epistasis and down, there are multiple paths through the space. With the addition of fourth-order epistasis, all paths but two become inaccessible. The addition of fifth-order epistasis opens the space up again, but to a different set of trajectories than what existed in the third-order space.

**Fig 5:**
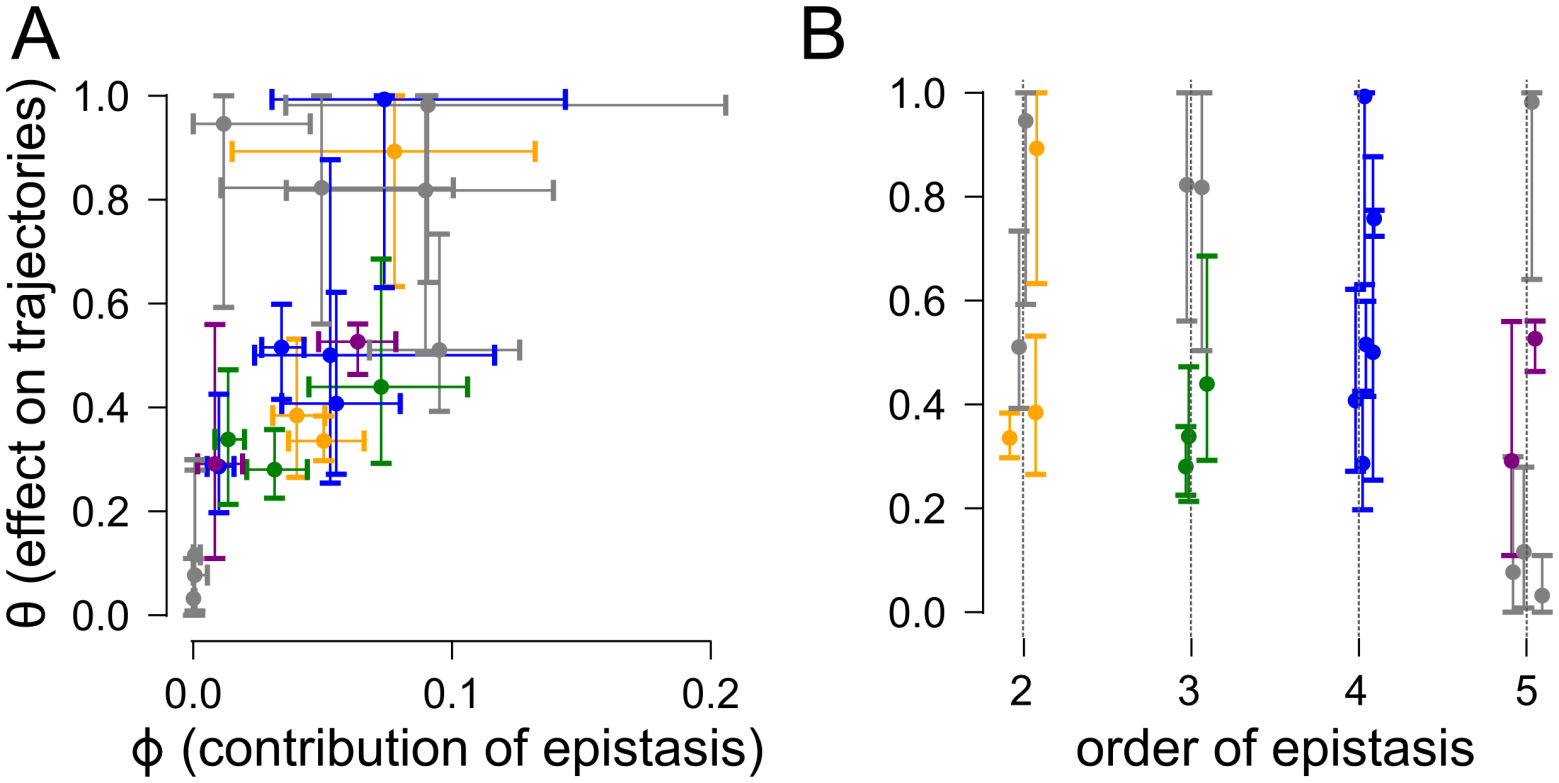
The magnitude of epistasis, not its order, predicts its effects on trajectories. Panel A shows *θ* graphed against *ϕ* for all datasets. Panel B shows *θ* graphed against the order of epistasis for all datasets. Points are colored by the order of epistasis: second (orange), third (green), fourth (blue), and fifth (purple). Error bars are 95% confidence intervals. Gray points are orders that could not be distinguished from experimental uncertainty (*p* < 0.05).

We next asked whether magnitude of epistasis or the order of epistasis was a stronger predictor of its effect on evolutionary trajectories. We plotted *ϕ* versus *θ* for each order for each dataset on a single plot (Fig 5A). This reveals a correlation between the magnitude of the epistasis and its effect on trajectories. In contrast, we see no correlation between the order of epistasis and its effect on evolutionary trajectories (Fig 5B). When epistasis contributes more than ≈5% of the variation in fitness, regardless of order, the divergence in trajectory probabilities with and without the epistasis is 40% or greater. The magnitude of epistasis—not its order—predicts its effect.

### High-order epistasis limits evolutionary predictability

Our next question was more practical: how important is epistasis for predicting evolutionary trajectories in these datasets? We imagined an experiment in which we measured the effects of all mutations in the ancestral genotype. We then asked if we could take these individually measured mutational effects and predict evolutionary trajectories.

To ask this question, we re-analyzed the epistasis present in all six datasets, this time using the ancestral genotype as the reference state. In this formulation, the first-order coefficients are the effect of each mutation by itself in the ancestral background, the second-order coefficients are the difference in the effects of mutations introduced in pairs versus separately, and third-order coefficients are the difference in the fitness of genotypes combining three mutations versus two mutations that cannot be explained by the first- and second-order coefficients. (This has been called the “biochemical” or “local” model of high-order epistasis [12].) We describe this further in the Materials & method section.

**Fig 6:**
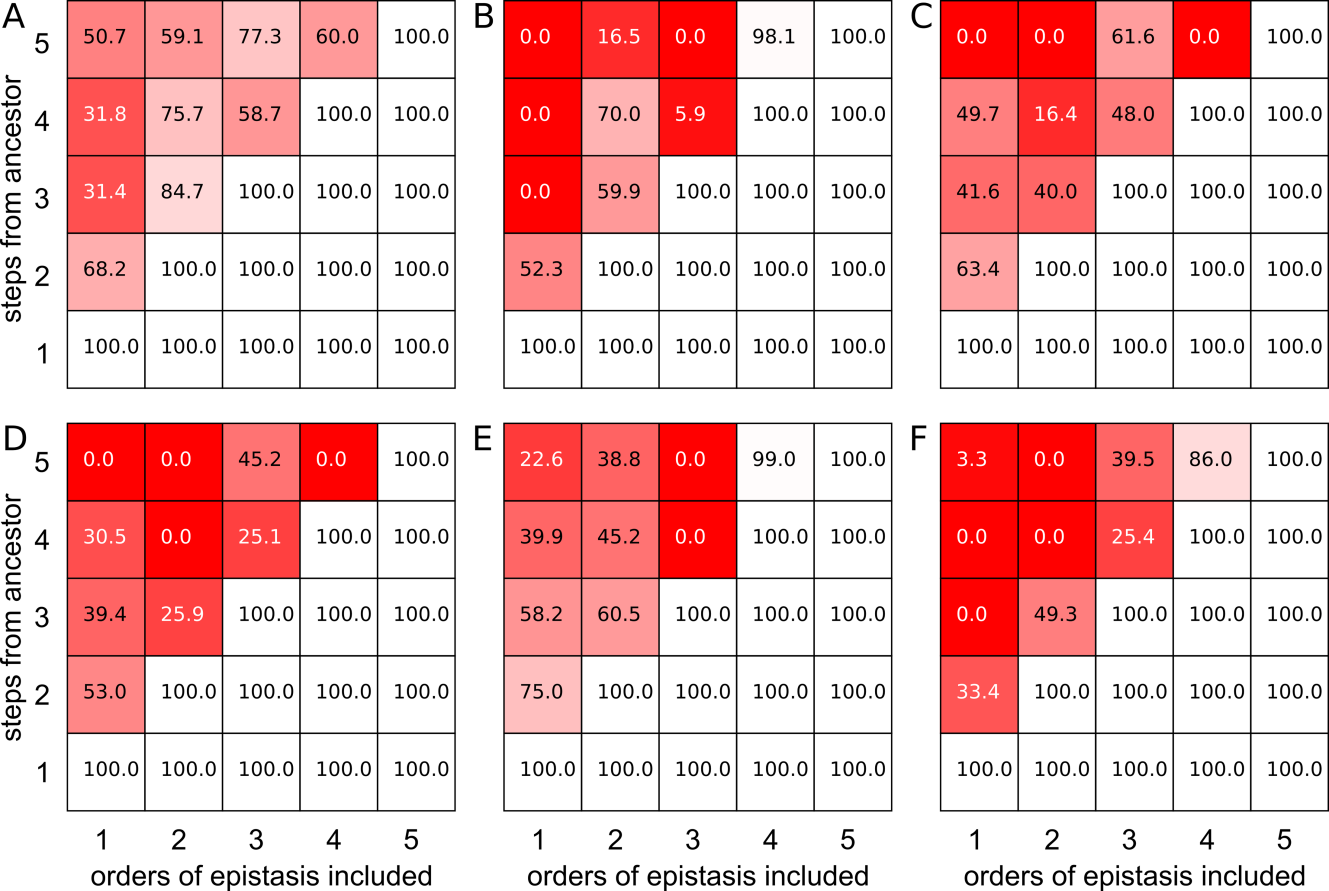
Epistasis complicates predicting trajectories from the ancestral genotype. Panels show trajectory prediction accuracy (color) for different amounts of pistasis included in the model (x-axis) and for different length trajectories away from the ancestral genotype (y-axis). Accuracy is measured as the difference in the probability distributions for trajectories through the truncated and original maps, ranging from 0.0% (red, poor accuracy) to 100% (white, perfect accuracy). Panels A-F correspond to datasets I-VI.

To characterize the effect of epistasis on our ability to predict evolutionary trajectories of increasing length, we calculated the probability of all possible forward trajectories of a defined number of steps starting from the ancestral genotype, and then repeated this probability calculation using maps truncated to various orders of epistasis. The difference in the actual and truncated map trajectory probability distributions measures our predictive power for evolutionary trajectories. We show these results in Fig 6 for all six datasets. In each panel, we plot inclusion of increasing orders of epistasis left-to-right (starting from additive and going to fifth-order) and increasing trajectory length bottom-to-top (starting from one-step and going to five-step). The overlap between the trajectory distribution for the truncated and real map for each epistasis/trajectory-length is shown as a color ranging from white (perfect prediction) to red (poor prediction).

We found that all orders of epistasis were important for predicting evolutionary trajectories. Dataset IV (panel D) illustrates behavior seen across all datasets, so we will use it as a specific example. In this dataset, additive coefficients are inadequate to capture even two-step trajectories: the trajectory probability distribution for two-step mutations only overlaps by 53.0% for the truncated and real maps. The prediction gets worse for longer trajectories, dropping to 39.4% for three steps, 30.5% for four steps, and 0.0% for five steps. The overlap for the final step is 0.0% because the additive model does not predict that the five-mutation genotype will be more fit than the four-mutation genotype. Trajectories in the additive map therefore do not proceed to this final genotype.

Adding pairwise epistasis to the model allows perfect “prediction” of the two-step trajectories, as we have perfect knowledge of the fitness values of all possible single and double mutants. But the three-step and four-step trajectories are predicted worse with pairwise epistasis included than with the additive map. The three-step overlap is 25.9%, while the four-step and five-step trajectory overlap is 0.0. The four-mutation and five-mutation genotypes are predicted to have low fitness. Adding third-order epistasis—now imagining that we characterized all possible single, double, and triple mutants in the ancestral genotype—allows us to “predict” trajectories up to three steps long; however, it fails for four- and five-step trajectories. The overlap is 25.1% and 45.2% respectively. Even the addition of fourth-order epistasis is insufficient to capture the five-step trajectories: the overlap for five-step trajectories is 0.0%.

Dataset IV is a particularly clean example, but all six datasets exhibit similar behavior (Fig 6). Neglecting epistasis leads to poor predictions of trajectories starting from the ancestral genotype. The lower-order the truncation, the worse the prediction as more mutations accumulate. Third- and fourth-order epistasis had an appreciable effect on all datasets. Fifth-order epistasis had an effect in four of the six datasets. Like the analysis using the global model above, high-order epistasis relative to the ancestral genotype potently alters evolutionary trajectories.

## Discussion

Our analysis reveals that high-order epistasis can strongly shape evolutionary trajectories. Removal of three-, four-, and five-way interactions between mutations significantly alters the probabilities of trajectories through genotype-fitness maps (Figs 2 and 4). This result is robust to uncertainty in the measured fitness values (Fig 3) and appears to be a general pattern in many maps (Fig 5). Finally, neglecting high-order epistasis leads to poor predictions of evolutionary trajectories through these maps (Fig 6).

In the majority of datasets, low-order models provide useful estimates of fitness. For datasets I-IV, ignoring three-way and higher-interactions yields fitness values within 15% of the actual map (Fig 1B). Dataset I would be particularly close, yielding fitness values within 2.5% of the actual map. This is consistent with other analyses of high-order epistasis in other datasets, which suggest that additive and pairwise epistatic effects can often provide sufficient information to predict multi-mutation fitness values to within 5-10% [6, 9–15].

While low-order models can often describe fitness with some degree of precision, low-order models are inadequate to describe evolutionary trajectories in any of the datasets. Even in dataset I, third- and fourth-order interactions potently shape evolutionary trajectories. The probability distributions of trajectories with and without fourth-order epistasis differ by 29.2%. And, as the magnitude of epistasis increases, its effect on trajectories grows (Fig 5A). In some instances, addition of high-order interactions completely shifts the set of trajectories available (Fig 4).

The effect of high-order epistasis on evolutionary trajectories is profound. We can build this intuition by imagining predicting evolutionary trajectories. If we start with knowledge of the individual effects of mutations in the ancestral background we can predict the first move perfectly, but not the second move. Pairwise epistasis means the effect of the second mutation is modulated by the presence of the first. We might try to overcome this difficulty by measuring the effect of each mutation and each pair of mutations, thereby accounting for pairwise epistasis. But our results reveal this is still insufficient to predict trajectories past the second step. There are three-way interactions that alter the effect of the third mutation, even after accounting for the first- and second-order effects of mutations. This continues all the way to fifth-order in these five-site datasets.

This has two implications. First, this adds to the growing recognition of extensive contingency in evolution [3, 4, 8, 32]. The effect of an event today is contingent on a whole collection of previous events. Remarkably, we found that this contingency is mediated by epistasis at all orders, including up to five-way interactions between mutations. Second, this work implies that measuring the individual effects of many mutations in a single genetic background, despite revealing a local fitness landscape [6, 33, 34], will be of limited utility for understanding evolution past the first few moves.

We expect the effect of high-order epistasis on trajectories will be amplified in larger maps that have more mutations. In a larger map, more mutations compete for fixation—each modulated by high-order interactions with previous substitutions—leading to even greater contingency on specific substitutions that occurred in the past. Further, the small maps we studied artificially limit the effects of high-order epistasis, as larger maps could, potentially, have even higher-order interactions. But even if no epistasis above fifth-order is present, trajectories will have more steps in a larger map; therefore, a fifth-order interaction could alter the relative probabilities of many more future moves in a larger space.

One open question is the effect of recombination on this radical contingency. We studied trajectories in which mutations fixed sequentially. This means our results are directly applicable to asexual organisms and loci in tight linkage, such as mutations to individual genes. Once recombination comes into play, other dynamics become possible. While recombination can completely overcome pairwise epistasis [35], it is unclear whether this result will apply to higher-order interactions.

High-order epistasis appears to be a ubiquitous feature of experimental genotype-fitness (and genotype-phenotype) maps [6, 9–15]. The origins of this epistasis remain unknown. Further, epistasis may go to much higher-order than yet observed, leading to extremely long-term memory in evolution. The observation of cryptic epistasis between genetic backgrounds that appear similar, but in which mutations have radically different effects, may point to high-order epistasis between mutations in diverging backgrounds [33, 36]. Whatever the origins or order may be, our work reveals that combinations of early substitutions continue to have an effect as future mutations accumulate: the past continues to press upon the present.

## Materials and methods

### Removal of high-order epistasis

We used the following protocol to remove specific orders of epistasis from genotype-fitness maps. The steps correspond directly to the pipeline shown in S1 Fig, which is described in detail in Sailer et al. [15].

1. We identified an appropriate, possibly nonlinear, scale for the map by fitting a power transformation to the genotype-fitness map:
  $${\vec{F}}_{experimental}=\frac{{\left({\hat{\vec{F}}}_{add}+A\right)}^{\lambda }-1}{\lambda {\left(GM\right)}^{\lambda -1}}+B,$$ (1)
  where ${\vec{F}}_{experimental}$ is the vector of the observed fitness values, ${\hat{\vec{F}}}_{add}$ is the fitness of each genotype assuming each mutation has the same, average effect in all backgrounds, *A* and *B* are translation constants, *GM* is the geometric mean of $\left({\hat{\vec{F}}}_{add}+A\right)$, and λ is a scaling parameter. ${\hat{\vec{F}}}_{add}$ is given by:
  $$F_{add,i}=\sum_{j=1}^{j\leq L}\left\langle \Delta F_{j}\right\rangle x_{i,j}$$ (2)
  where 〈Δ*F*_*j*_〉 is the average effect of mutation *j* across all backgrounds, *x*_*i*,*j*_ is an index that encodes whether or not mutation *j* is present in genotype *i*, and *L* is the number of sites. We first regressed ${\hat{F}}_{add}$, and then regressed the power transform.
2. We linearized each map by transforming each element in ${\vec{F}}_{experimental}$ with the nonlinear scale and coefficients determined in step 1. For each element in ${\vec{F}}_{experimental}$, we performed:
  $$F_{linear}={\left\{{\hat{\lambda }\left(GM\right)}^{\lambda -1}\left(F_{experimental}-\hat{B}\right)+1\right\}}^{1/\hat{\lambda }}-\hat{A}.$$ (3)
3. We decomposed the variation in fitness into epistatic coefficients using a linear decomposition of the form:
  $$\vec{\beta }=X^{-1}{\vec{F}}_{linear},$$ (4)
  where $\vec{\beta }$ is a collection of epistatic coefficients (ranging from 0^th^ to *L*^*th*^ order) and **X** is a design matrix that indicates which coefficients contribute to fitness in which genotype. For most of the work described, we used a Hadamard matrix for **X**, which uses the geometric center of the genotype-fitness map as a reference state. [10, 12, 15, 17]. To construct this matrix, we encoded each mutation within each genotype as -1 (wildtype) or +1 (mutant) [12, 15]. For the final section, we use a “local” matrix for **X**, which measures the effect of each mutation relative to a defined reference phenotype. To construct this matrix, we encoded each mutation within each genotype as 0 (wildtype) or 1 (mutant). These to forms of **X** can be readily inter-converted [12].
4. We truncated epistasis from the linearized map by setting the epistatic coefficients from orders of interest to 0, creating ${\vec{\beta }}_{trunc}$.
5. We recalculated the linearized fitness values, with truncated epistasis by:
  $${\vec{F}}_{linear,trunc}=X{\vec{\beta }}_{trunc}.$$ (5)
6. We transformed the ${\vec{F}}_{linear,trunc}$ onto the original, nonlinear scale using Eq 1, with ${\vec{F}}_{linear,trunc}$ in place of ${\vec{F}}_{add}$.
7. We used the final ${\vec{F}}_{trunc}$ values to construct a genotype-fitness map in which orders of epistasis were selectively removed, leaving the global, nonlinear scale intact.

We quantified the contribution of epistasis to each map (*ϕ*) by determining the difference in the variation explained by the *i*^*th*^ and (*i* − 1)^*th*^ orders. $\phi =\rho_{i}^{2}-\rho_{i-1}^{2}$, where $\rho_{x}^{2}$ is the squared Pearson coefficient between linear fitness values in a model truncated to order *x* (${\vec{F}}_{linear,trunc-to-x}$) and linear fitness values determined from the original map (${\vec{F}}_{linear}$).

### Evolutionary trajectories

We calculated the probability of a given evolutionary trajectory as series of independent, sequential fixation events. We assumed that the time to fixation for each mutation was much less than the time between mutations (the so-called strong selection/weak mutation regime) [1, 18, 19]. The relative probability of an evolutionary trajectory *i* is the product of its required fixation events relative to all possible trajectories:

$$p_{i}=\frac{\prod_{x\in S_{i}}\pi_{x\to x+1}}{\sum_{j\in T}\prod_{x\in S_{j}}\pi_{x\to x+1}},$$ (6)

where *π*_*x*→*x*+1_ is the fixation probability for genotype *x* + 1 in the *x* background, *S*_*i*_ is the set of steps that compose trajectory *i*, and *T* is the set of all forward trajectories. The model assumes the mutation rate is the same for all sites, and that population size and mutation rates are fixed over the evolutionary trajectory [1, 19–22]. We calculated *π*_*x*→*x*+1_ for each step using the Gillespie model [23]

$$\pi_{x\to x+1}=\frac{1-e^{-s_{x\to x+1}}}{1-e^{-Ns_{x\to x+1}}}=\frac{1-e^{-(1-w_{x+1}/w_{x})}}{1-e^{-N(1-w_{x+1}/w_{x})}},$$ (7)

where *N* is population size, *s* is the selection coefficient and *w*_*x*_ and *w*_*x*+1_ are the relative fitnesses of the *x* and *x* + 1 genotypes visited over the trajectory.

To determine the difference between sets of trajectories in maps with and without high-order epistasis, we measured the magnitude of the difference in probability for all *L*! forward trajectories through each space. We did so by:

$$\theta =\frac{\sum_{i=1}^{i=L!}\left|p_{i}^{experimental}-p_{i}^{trunc}\right|}{2},$$ (8)

where $p_{i}^{experimental}$ is the probability of the *i*^*th*^ trajectory within the experimental map and $p_{i}^{trunc}$ is the probability of that same trajectory in a truncated map, with high-order epistasis removed.

### Software

We implemented the epistasis and trajectory models using Python 3 extended with the *numpy* and *scipy* packages [24]. We used the python package *scikit-learn* to perform linear regression with truncated forms of these models [25]. Plots were generated using *matplotlib* and *jupyter* notebooks [26, 27]. Our full software package is available in the *epistasis* package via github (https://harmslab.github.com/epistasis).

## Supporting information

S1 JSON Text file containing individual replicate fitness measurements for each genotype in the six experimental datasets. (JSON)

S1 Fig Flow chart for removing epistasis in a genotype-fitness map. This chart describes the pipeline we used to truncate epistasis from genotype-fitness maps. The data shown are for dataset II. Networks (left) show all 2^5^ genotypes, arranged from ancestral (top) to derived (bottom), colored by relative fitness from 1.0 (purple) to 1.30 (yellow). The correlation plots (middle) show the fitness of each genotype plotted against the fitness of that genotype assuming each mutation has a linear, additive effect on fitness (*F*_*add*_). Y-axes correspond to: the experimentally measured fitness (*F*_*experimental*_, panel 2); the experimentally measured fitness linearized using the red scale in panel 2 (*F*_*linear*_, panel 3); fitness values with third-, fourth- and fifth-order epistasis removed, on the linear scale from panel 3 (*F*_*linear*,*trunc*_, panel 5); and fitness values with truncated epistasis on the red nonlinear scale from panel 2 (*F*_*trunc*_, panel 6). The right-most panels show the fraction of variation explained by first- (red), second- (orange), third- (green), and fourth-order (purple) epistatic coefficients. The area occupied by each color indicates its contribution to the fitness on the linear scale.
(TIF)

S2 Fig Schematic of our resampling protocol. Two-mutation maps are shown throughout, colored by fitness from low (purple) to high (yellow). We sampled from two maps: the original map with uncertainty (A, red) and a “null” map in which epistasis was removed, but experimental uncertainty maintained (B, blue). We used the same sampling protocol on each (“Start”). We generated pseudoreplicates (*s*_1_, *s*_2_, … *s*_*n*_) from uncertainty (Gaussian curves above the color spectrum in A and B). We then truncated the pseudoreplicate to *i*^*th*^ and (*i* − 1)^*th*^ order epistasis and calculated *ϕ* and *θ* for each pseudoreplicate: {(*ϕ*_1_, *θ*_1_), (*ϕ*_2_, *θ*_2_), … (*ϕ*_*n*_, *θ*_*n*_)}. We can then plot and compare these distributions on *ϕ*/*θ* axes.
(TIF)

S3 Fig Epistasis alters trajectories in dataset I. A) Colors, panel layouts, and statistics are as in Fig 2. B) Colors, panel layouts, and statistics are as in Fig 1A. C-D): Colors and panel layouts are as in Fig 3.
(TIF)

S4 Fig Epistasis alters trajectories in dataset III. A) Colors, panel layouts, and statistics are as in Fig 2. B) Colors, panel layouts, and statistics are as in Fig 1A. C-D): Colors and panel layouts are as in Fig 3.
(TIF)

S5 Fig Epistasis alters trajectories in dataset III. A) Colors, panel layouts, and statistics are as in Fig 2. B) Colors, panel layouts, and statistics are as in Fig 1A. C-D): Colors and panel layouts are as in Fig 3.
(TIF)

S6 Fig Epistasis alters trajectories in dataset IV. A) Colors, panel layouts, and statistics are as in Fig 2. B) Colors, panel layouts, and statistics are as in Fig 1A. C-D): Colors and panel layouts are as in Fig 3.
(TIF)

S7 Fig Epistasis alters trajectories in dataset V. A) Colors, panel layouts, and statistics are as in Fig 2. B) Colors, panel layouts, and statistics are as in Fig 1A. C-D): Colors and panel layouts are as in Fig 3.
(TIF)

S8 Fig Epistasis alters trajectories in dataset VI. A) Colors, panel layouts, and statistics are as in Fig 2. B) Colors, panel layouts, and statistics are as in Fig 1A. C-D): Colors and panel layouts are as in Fig 3.
(TIF)
