# Supplementary figures and images for: High-order epistasis shapes evolutionary trajectories
Source: PLoS Comput Biol. 2017 May 15;13(5):e1005541. doi: 10.1371/journal.pcbi.1005541 (PMC5448810; doi:10.1371/journal.pcbi.1005541)

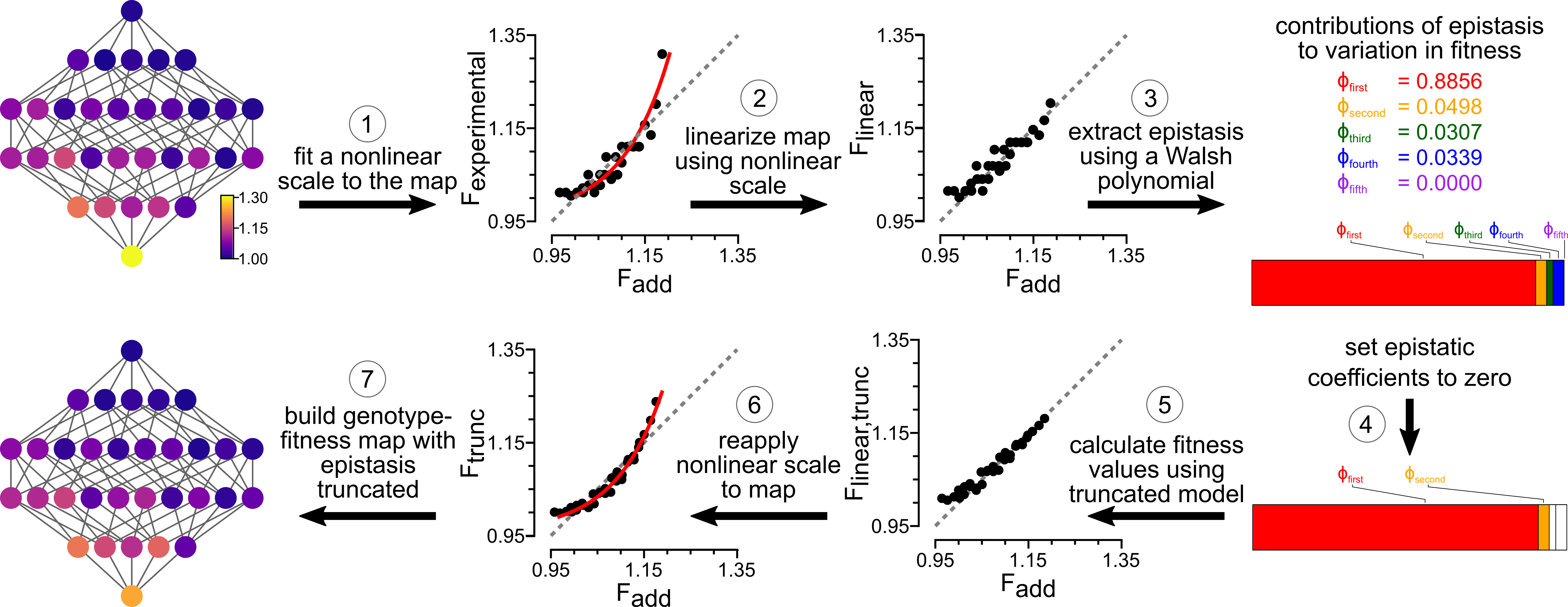

Supplement: S1 Fig — This chart describes the pipeline we used to truncate epistasis from genotype-fitness maps. The data shown are for dataset II. Networks (left) show all 25 genotypes, arranged from ancestral (top) to derived (bottom), colored by relative fitness from 1.0 (purple) to 1.30 (yellow). The correlation plots (middle) show the fitness of each genotype plotted against the fitness of that genotype assuming each mutation has a linear, additive effect on fitness (Fadd). Y-axes correspond to: the experimentally measured fitness (Fexperimental, panel 2); the experimentally measured fitness linearized using the red scale in panel 2 (Flinear, panel 3); fitness values with third-, fourth- and fifth-order epistasis removed, on the linear scale from panel 3 (Flinear,trunc, panel 5); and fitness values with truncated epistasis on the red nonlinear scale from panel 2 (Ftrunc, panel 6). The right-most panels show the fraction of variation explained by first- (red), second- (orange), third- (green), and fourth-order (purple) epistatic coefficients. The area occupied by each color indicates its contribution to the fitness on the linear scale. (TIF) [file pcbi.1005541.s002.tif]

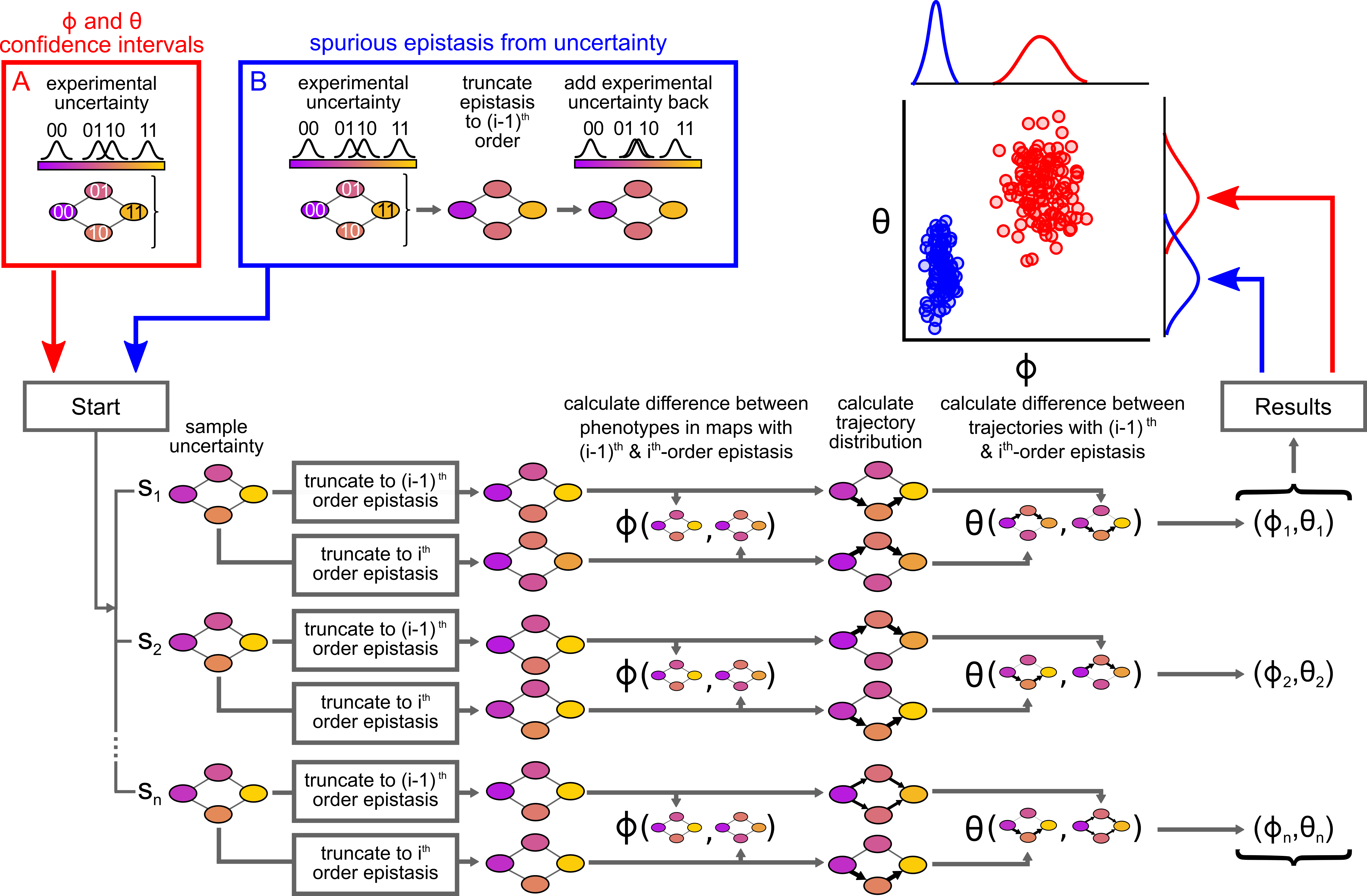

Supplement: S2 Fig — Two-mutation maps are shown throughout, colored by fitness from low (purple) to high (yellow). We sampled from two maps: the original map with uncertainty (A, red) and a “null” map in which epistasis was removed, but experimental uncertainty maintained (B, blue). We used the same sampling protocol on each (“Start”). We generated pseudoreplicates (s1, s2, … sn) from uncertainty (Gaussian curves above the color spectrum in A and B). We then truncated the pseudoreplicate to ith and (i − 1)th order epistasis and calculated ϕ and θ for each pseudoreplicate: {(ϕ1, θ1), (ϕ2, θ2), … (ϕn, θn)}. We can then plot and compare these distributions on ϕ/θ axes. (TIF) [file pcbi.1005541.s003.tif]

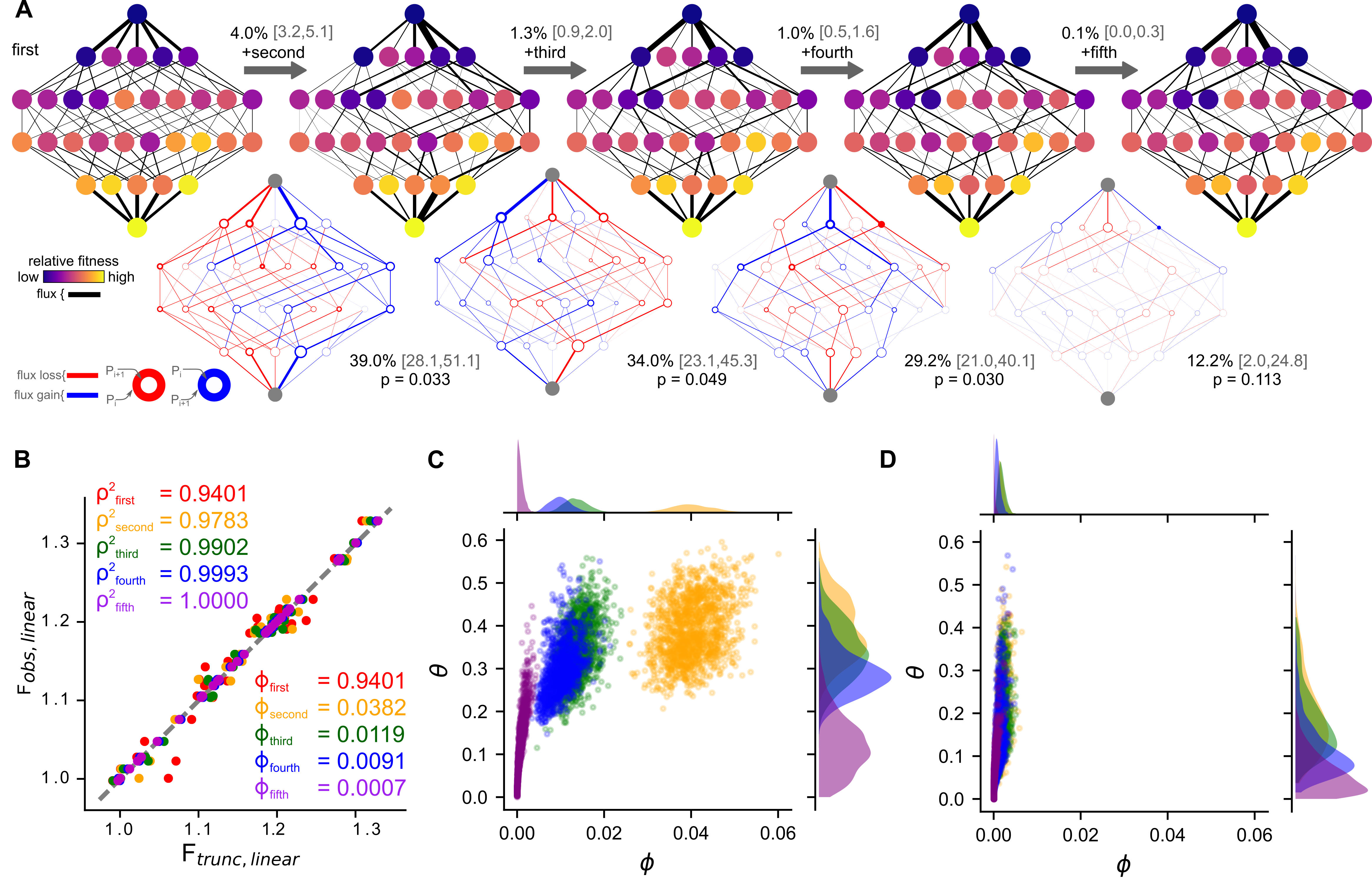

Supplement: S3 Fig — A) Colors, panel layouts, and statistics are as in Fig 2. B) Colors, panel layouts, and statistics are as in Fig 1A. C-D): Colors and panel layouts are as in Fig 3. (TIF) [file pcbi.1005541.s004.tif]

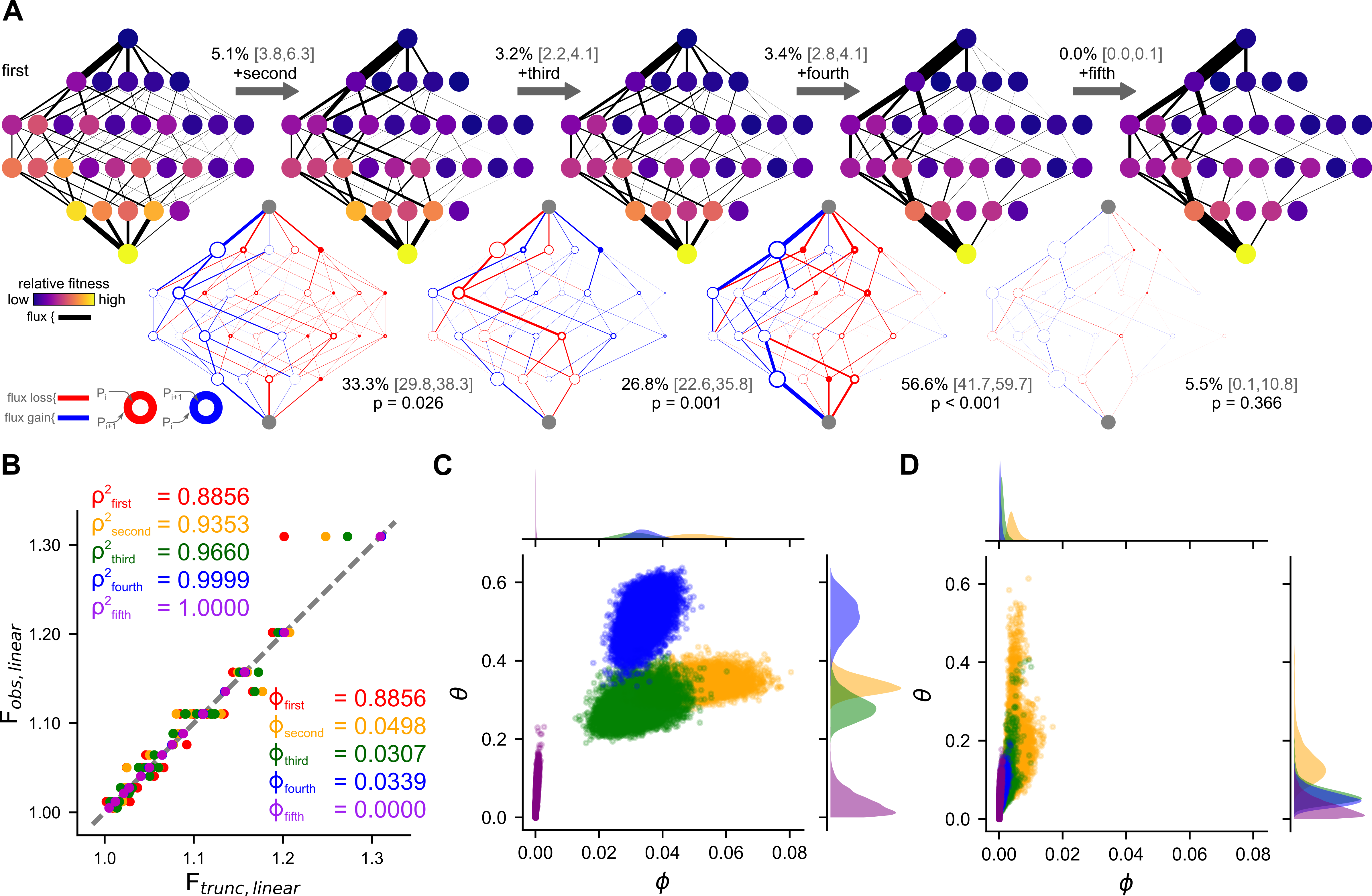

Supplement: S4 Fig — A) Colors, panel layouts, and statistics are as in Fig 2. B) Colors, panel layouts, and statistics are as in Fig 1A. C-D): Colors and panel layouts are as in Fig 3. (TIF) [file pcbi.1005541.s005.tif]

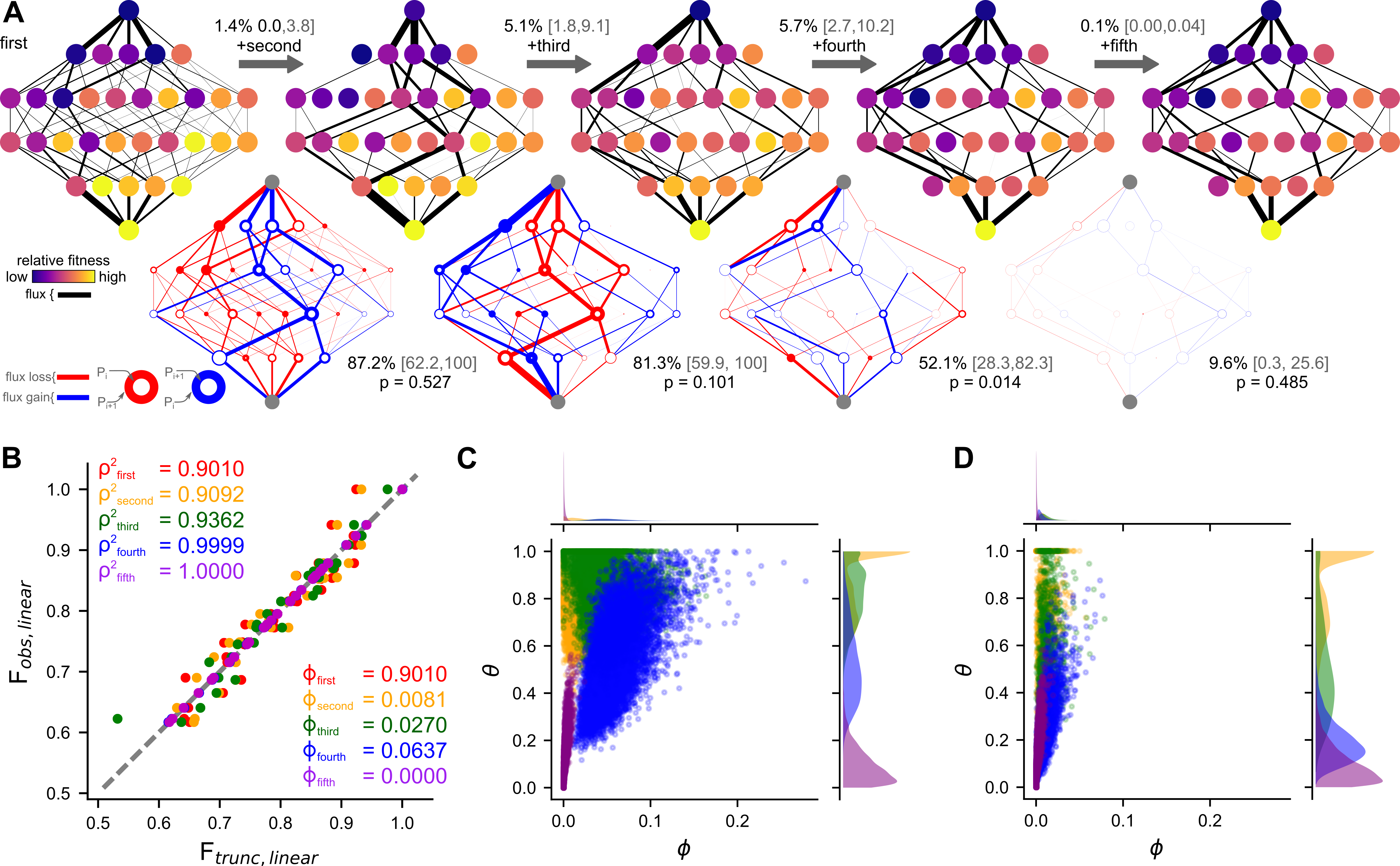

Supplement: S5 Fig — A) Colors, panel layouts, and statistics are as in Fig 2. B) Colors, panel layouts, and statistics are as in Fig 1A. C-D): Colors and panel layouts are as in Fig 3. (TIF) [file pcbi.1005541.s006.tif]

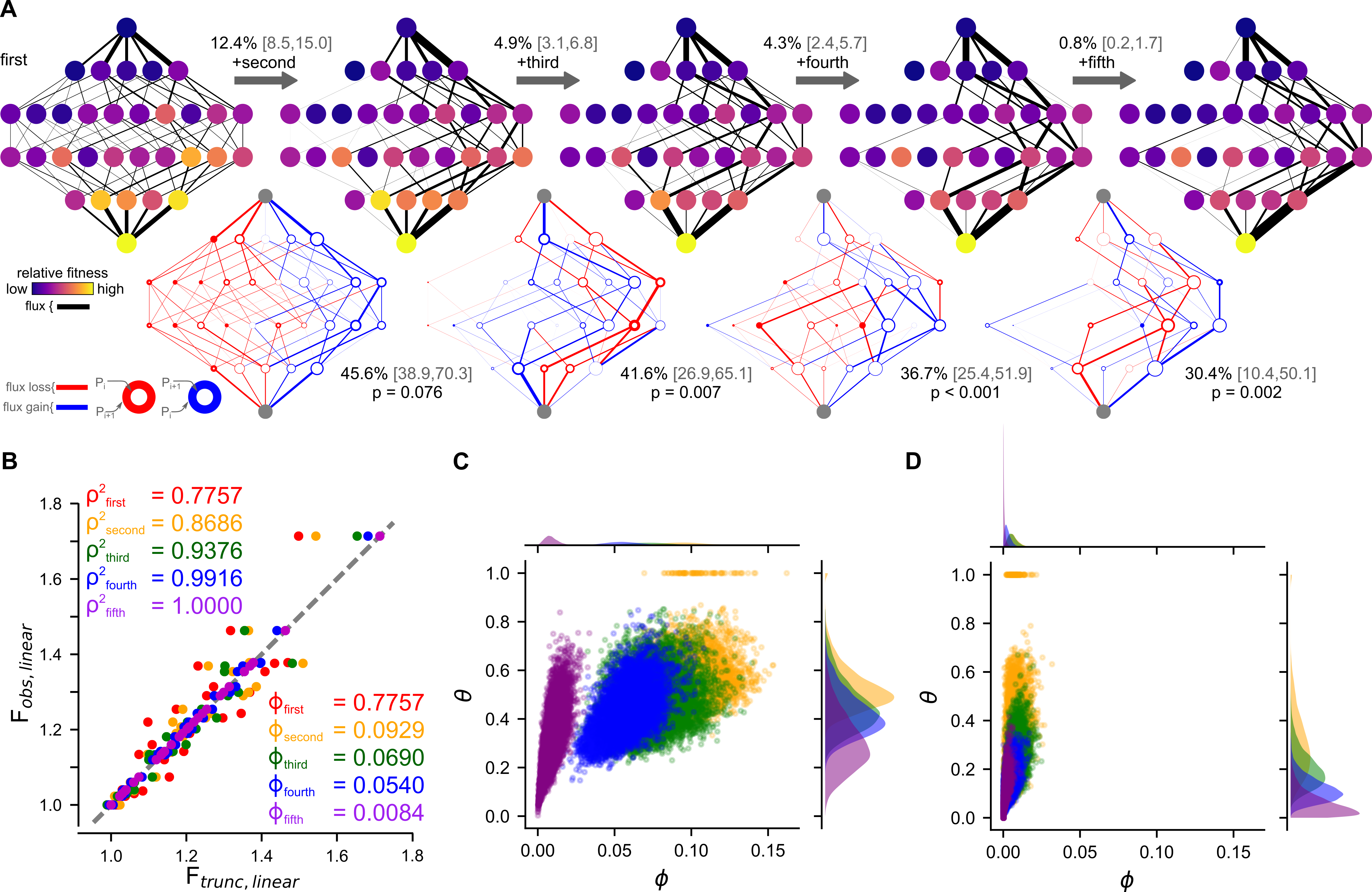

Supplement: S6 Fig — A) Colors, panel layouts, and statistics are as in Fig 2. B) Colors, panel layouts, and statistics are as in Fig 1A. C-D): Colors and panel layouts are as in Fig 3. (TIF) [file pcbi.1005541.s007.tif]

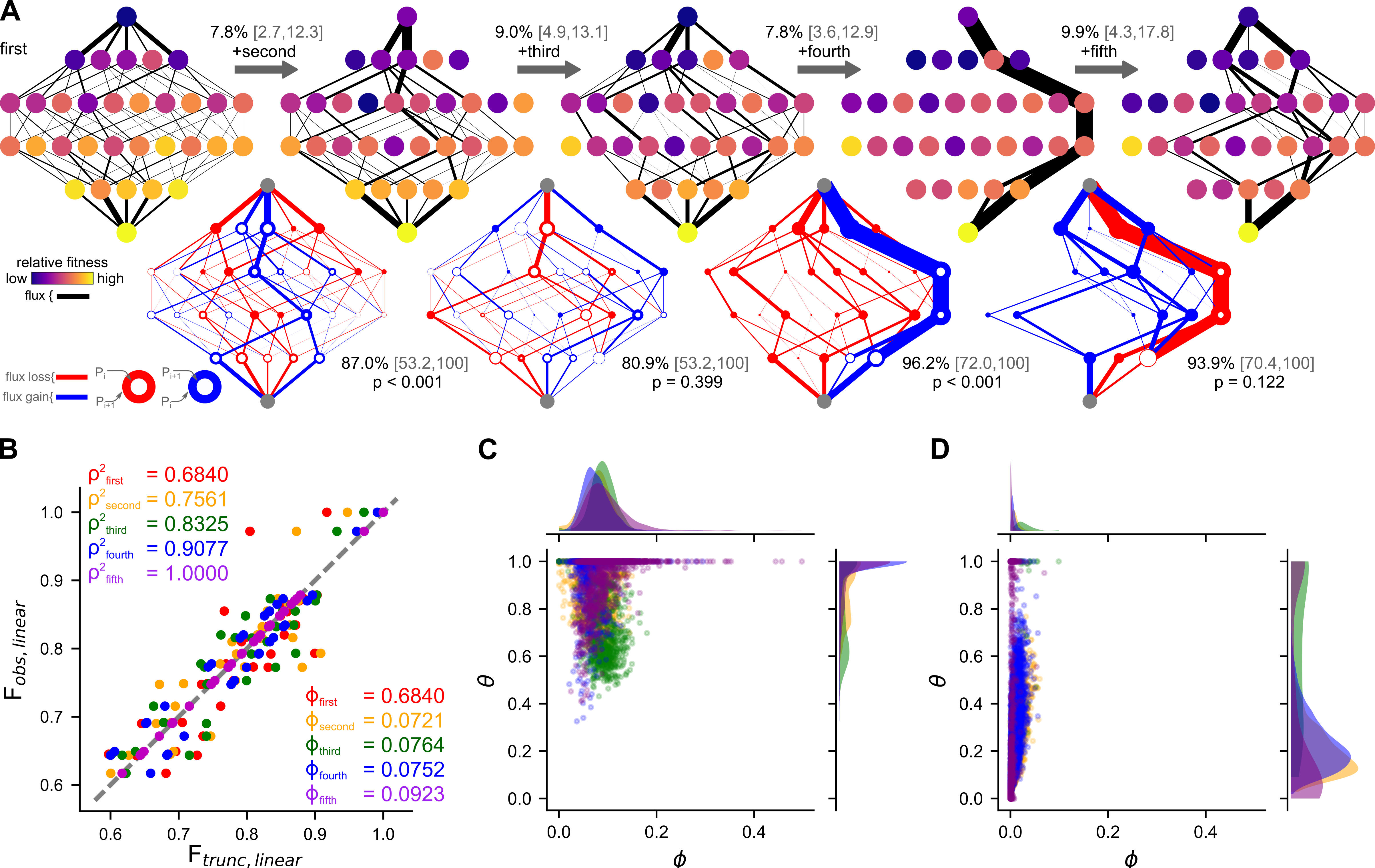

Supplement: S7 Fig — A) Colors, panel layouts, and statistics are as in Fig 2. B) Colors, panel layouts, and statistics are as in Fig 1A. C-D): Colors and panel layouts are as in Fig 3. (TIF) [file pcbi.1005541.s008.tif]

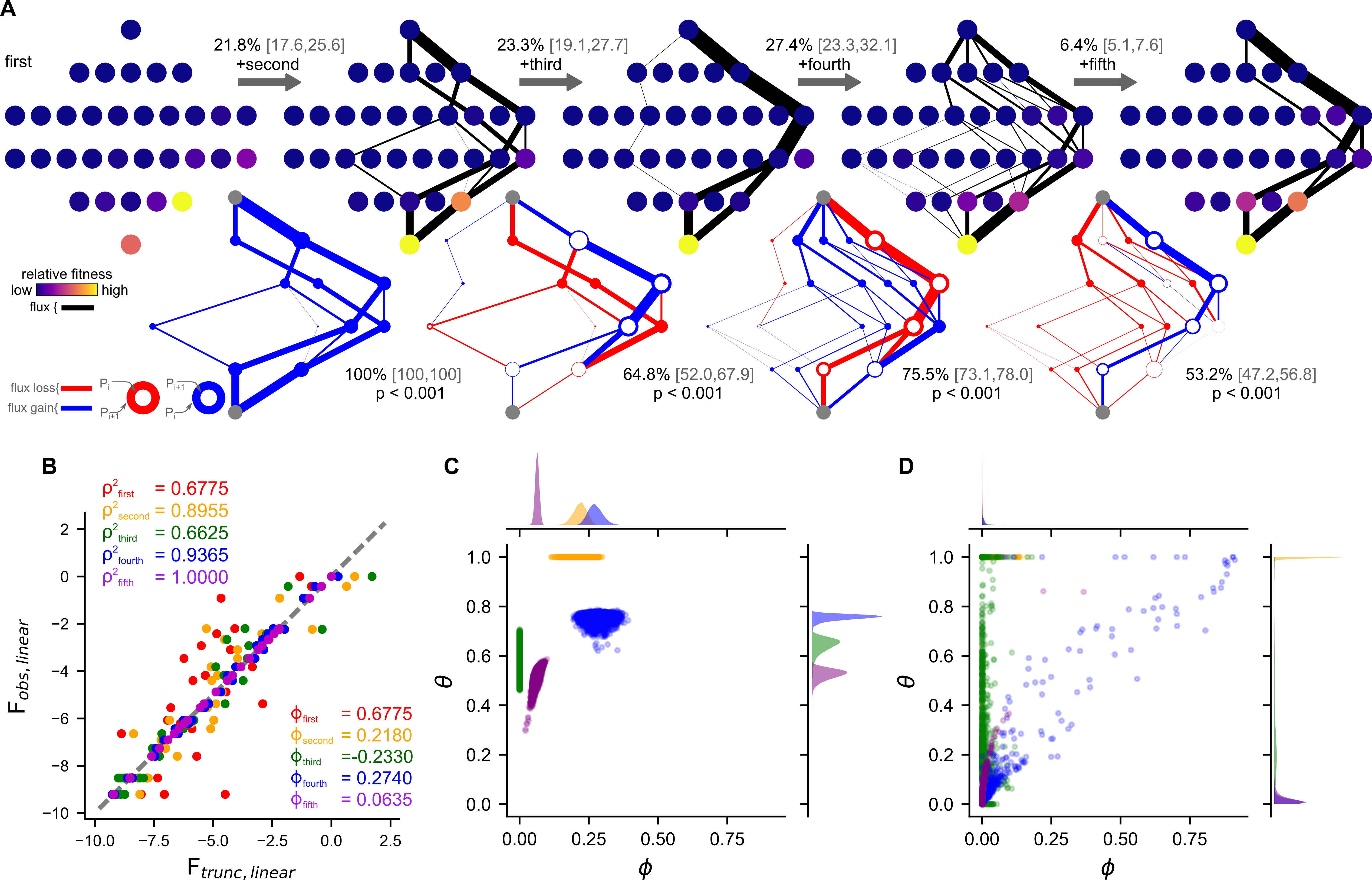

Supplement: S8 Fig — A) Colors, panel layouts, and statistics are as in Fig 2. B) Colors, panel layouts, and statistics are as in Fig 1A. C-D): Colors and panel layouts are as in Fig 3. (TIF) [file pcbi.1005541.s009.tif]
